# Antibiotic Resistance in Agricultural Soil and Crops Associated to the Application of Cow Manure-Derived Amendments From Conventional and Organic Livestock Farms

**DOI:** 10.3389/fvets.2021.633858

**Published:** 2021-02-23

**Authors:** Leire Jauregi, Lur Epelde, Itziar Alkorta, Carlos Garbisu

**Affiliations:** ^1^Department of Conservation of Natural Resources, NEIKER – Basque Institute for Agricultural Research and Development, Basque Research and Technology Alliance (BRTA), Derio, Spain; ^2^Department of Biochemistry and Molecular Biology, University of the Basque Country (UPV/EHU), Bilbao, Spain

**Keywords:** emerging contaminants, mobile genetic elements, organic farming, soil microbial diversity, antibiotic resistance genes

## Abstract

The application of organic amendments to agricultural soil can enhance crop yield, while improving the physicochemical and biological properties of the recipient soils. However, the use of manure-derived amendments as fertilizers entails environmental risks, such as the contamination of soil and crops with antibiotic residues, antibiotic resistance genes (ARGs) and mobile genetic elements (MGEs). In order to delve into these risks, we applied dairy cow manure-derived amendments (slurry, fresh manure, aged manure), obtained from a conventional and an organic farm, to soil. Subsequently, lettuce and wheat plants were grown in the amended soils. After harvest, the abundance of 95 ARGs and MGE-genes from the amended soils and plants were determined by high-throughput qPCR. The structure of soil prokaryotic communities was determined by 16S rRNA amplicon sequencing and qPCR. The absolute abundance of ARGs and MGE-genes differed between treatments (amended vs. unamended), origins of amendment (conventional vs. organic), and types of amendment (slurry vs. fresh manure vs. aged manure). Regarding ARG-absolute abundances in the amendments themselves, higher values were usually found in slurry vs. fresh or aged manure. These abundances were generally higher in soil than in plant samples, and higher in wheat grain than in lettuce plants. Lettuce plants fertilized with conventional amendments showed higher absolute abundances of tetracycline resistance genes, compared to those amended with organic amendments. No single treatment could be identified as the best or worst treatment regarding the risk of antibiotic resistance in soil and plant samples. Within the same treatment, the resistome risk differed between the amendment, the amended soil and, finally, the crop. In other words, according to our data, the resistome risk in manure-amended crops cannot be directly inferred from the analysis of the amendments themselves. We concluded that, depending on the specific question under study, the analysis of the resistome risk should specifically focus on the amendment, the amended soil or the crop.

## Introduction

Antibiotics are indispensable tools for the treatment of bacterial infections in human medicine and veterinary medicine. Antibiotics are mainly used for the curative and, to a lesser extent, preventive treatment of bacterial infectious diseases. Besides, they are also used in many countries as growth promoters in animal production farms (1). However, the use of antibiotics for disease prevention is not recommended by the Word Health Organization (2) and the European Union banned the use of antibiotics for animal growth promotion in 2006 [Regulation (EC) No. 1831/2003]. The use, abuse and inappropriate use of antibiotics (i) in livestock farms for animal production purposes, (ii) in human medicine for the treatment of bacterial infections, and (iii) in agriculture for crop production purposes is gradually causing the emergence and dissemination of antibiotic resistant bacteria (some of them show simultaneous resistance to many—*multiresistant—*or even all—*panresistant—*known antibiotics), due to the selective pressure exerted by antibiotics on exposed bacterial populations. Many antibiotics used in veterinary practice are the same used to treat bacterial infections in humans or have the same mode of action or belong to the same antibiotic family (3), leading to the alarming intensification and augmentation of the well-known huge problem of multiresistant bacterial strains currently putting at risk, at a global scale, our capacity to fight and control bacterial human pathogens (4).

Most antibiotics administered to livestock are not fully metabolized and, hence, are released, together with their transformation products, into the environment along with the feces and urine (5). In fact, a considerable percentage (30–90%) of the antibiotic administered to a given animal for veterinary purposes can be directly excreted in the urine and feces (5). Animal manure is therefore a source of antibiotic contamination (antibiotics are nowadays considered emerging contaminants) and a reservoir of antibiotic resistant bacteria (ARB) harboring and potentially spreading antibiotic resistance genes (ARGs) (6). Animal manure is commonly applied to agricultural soil as organic fertilizer. Apart from providing valuable plant nutrients that can enhance crop yield, the application of manure can simultaneously improve soil physicochemical and biological properties, i.e., soil quality (7–9). Regrettably, the agronomic application of manure can also lead to the emergence and dissemination of ARB and ARGs in the amended agricultural soil and, subsequently, in the food crops grown for human consumption (10, 11). To make matters worse, ARB can disseminate ARGs to other bacteria through horizontal gene transfer (HGT) mediated by mobile genetic elements (MGEs), such as integrons, phages, plasmids, integrative conjugative elements, transposons, etc. (12, 13).

Understandably, most of the attention given to the problem of antibiotic resistance (AR) has been directed to hospital settings. Nonetheless, in the last years, more and more awareness is being developed concerning the vastly complex environmental dimension of AR and its central role in the emergence, maintenance and spread of AR at a global scale (14). Undeniably, the emergence and dissemination of AR in agroecosystems, resulting from the application of animal manure as organic fertilizer, begets a potential risk for human health and the environment, being currently an issue of much global concern that, urgently, requires the development and implementation of practices and management measures that mitigate (or, better, eliminate), such a risk (15). Among other measures aimed at enhancing the sustainability of animal production practices, organic livestock farming promotes a considerable reduction of the use of antibiotics for veterinary purposes, compared to conventional livestock systems. In principle, this reduction in antibiotic use implies concomitantly a lower level of selective pressure for bacterial populations to acquire and maintain AR by evolutionary adaptation mechanisms (16). In addition, the composting of animal manure has recurrently been reported as an effective option for the reduction of antibiotic concentrations in animal manure and, to a lesser extent, for the decrease in the abundance of ARGs in these animal-derived organic amendments (17, 18).

On the other hand, the presence of antibiotics and their transformation products (some of these are also bioactive compounds) in animal manure may significantly alter the composition of soil microbial communities when applied to agricultural soil. These antibiotic-induced changes in soil microbial composition frequently have important consequences for the soil resistome and mobilome (19, 20). Relevantly, soil microbial diversity (in terms of richness, evenness, composition, etc.) is regularly used as a biological indicator of the impact of disturbances (e.g., contamination) on soil health (21–23).

Our objective was to study, under controlled microcosm conditions, the emergence and dissemination of AR in agricultural soil and food crops (lettuce and wheat) derived from the application of dairy cow wastes as organic fertilizer. In order to delve into possible management practices that could minimize the resistome risk, we compared the effects of the application of: (i) three types of commonly used amendments: slurry vs. fresh manure vs. aged manure; and (ii) amendments from a conventional livestock farm vs. an organic livestock farm. To quantify the magnitude of the resistome risk in agricultural soil and food crops, we used the following end-points: (i) antibiotic concentrations; (ii) abundance of ARGs and MGE-genes in soils and plants (lettuce and wheat grain); and (iii) observed relationships between the structural diversity of soil prokaryotic communities (from 16S rRNA amplicon sequencing data) and the abundance of ARGs and MGE-genes. We hypothesized that the resistome risk will be higher in soils and plants: (i) amended with dairy cow wastes, compared to non-amended controls; (ii) amended with dairy cow wastes from the conventional livestock farm vs. the organic livestock farm; and (iii) amended with slurry wastes vs. fresh and aged wastes. We also hypothesized that lettuce samples will show a higher resistome risk than wheat grain samples.

## Materials and Methods

### Experimental Design

The amendments used in this study were kindly provided by two dairy cow farms located in the province of Biscay (Spain): a conventional livestock farm and an organic livestock farm. Three types of amendments (i.e., slurry, fresh manure, aged manure) from these two different origins (i.e., conventional livestock farm and organic livestock farm) were studied. In both farms, representative samples of cow slurry were taken from a pool where the feces and urine from the cows in production were deposited. In contrast, fresh manure samples were taken from the cow bedding (made from feces, urine, and wheat straw) of the non-producing cows: heifers, dry cows and cows undergoing treatment (the latter only in the conventional farm). As for the aged manure, a composite sample was taken from a manure pile that had been stored for ~6 months. All samplings were carried out on the same day. Fresh and aged manure samples were collected in polyethylene bags, while slurry samples were contained in plastic barrels. All samples were immediately transferred to the laboratory and stored at 4°C until use. The experimental soil was collected from the upper 30 cm layer of a semi-natural grassland field which, to our knowledge, has never been amended with any kind of inorganic or organic fertilizer. Immediately after collection, the soil was sieved to <4 mm. For our microcosm study, experimental pots containing 2 and 4 kg of dry weight (DW) soil were used for lettuce and wheat plants, respectively. The dose of amendment was carefully adjusted in order to provide an equivalent of 100 and 180 kg N ha^−1^ for lettuce and wheat plants, respectively. The amendments were manually incorporated into the soil and thoroughly mixed for homogenization purposes. A 2 week stabilization period was allowed before crop planting (lettuce seedlings) or sowing (wheat seeds). Lettuce (*Lactuca sativa* L. var. Batavia) and hard winter wheat (*Triticum aestivum* L. var. Qualidu) plants were used in this study, since they are most commonly grown in our region for agricultural purposes. Our experiment was carried out in a growth chamber under the following controlled conditions: 14/10 h light/dark cycle, 20/16°C day/night temperature, 70% relative humidity, and a photosynthetic photon flux density of 150 μmol photon m^−2^ s^−1^. Throughout the experimental period, plants were bottom watered every 2–3 days. Each treatment was replicated four times. Lettuce plants were harvested after 44 days of growth, while wheat plants were harvested after 171 days. For the determination of crop production, lettuce plants (aerial part = shoot biomass) were cut from the base with a scalpel and then freshly weighed. Similarly, in wheat plants, spikes were husked, and wheat grains freshly weighed. Dry weight of lettuce plants and wheat grains was determined by drying in an oven at 70°C until reaching a constant mass. On the other hand, soil samples were collected from the pots at crop harvest time (see below section Effect of Treatments on Biological Parameters Related to the Resistome Risk).

### Amendment and Soil Physicochemical Characterization

Before the beginning of the experiment, the dairy cow manure-derived amendments and the experimental semi-natural grassland soil were physicochemically characterized (24) according to the following parameters: pH, organic matter (OM) content, total nitrogen (N), potassium (K^+^), and Olsen phosphorus (P). Dry weight of soils was determined by drying in an oven at 30°C until reaching a constant mass. Mineral and pseudo-total metal concentrations were determined by Inductively Coupled Plasma Atomic Emission Spectrometry (ICP-AES) following aqua regia digestion (25). Antibiotic concentrations were determined by Liquid Chromatography Tandem Mass Spectrometry (LC-MS/MS) in SAILab Instrumental Analytical Solutions (Barcelona, Spain). In particular, the concentration of 57 antibiotics belonging to nine families (aminoglycosides, cephalosporins, macrolides, nitrofurans, penicillins, polypeptides, quinolones, sulphonamides, and tetracyclines) was quantified. For confirmation purposes and in order to assess the rate of degradation of the antibiotics present in the manure and soil samples, a second analysis of antibiotic concentrations was carried out 2 months later. In this second analysis, the antibiotic families that, in the first analysis, exceeded the detection limit of the technique in at least one of the studied antibiotics (i.e., polypeptides and quinolones) were again analyzed.

### Effect of Treatments on Biological Parameters Related to the Resistome Risk

For the assessment of the effect of treatments on biological parameters that provide information on resistome risk, at crop harvest time, soil samples were collected from the experimental pots and then sieved to <2 mm. Prior to DNA extraction, soil samples were washed twice in 120 mM K_2_PO_4_ (pH 8.0) to wash away extracellular DNA (26). DNA was extracted from soil samples (0.25 g DW soil) using the Power Soil^TM^ DNA Isolation Kit (MoBio Laboratories Inc., Carlsbad, CA). Similarly, DNA was extracted from plant samples using the innuPREP Plant DNA Kit (Analytik Jena, Jena, Germany). The concentration of soil and plant DNA was quantified with a NanoDrop spectrophotometer (ND-1000, Thermo Scientific, Wilmington, DE). Soil and plant DNA was stored at −20°C until use.

For the quantification of ARG and MGE-gene abundances, high-throughput real-time PCR (HT-qPCR) reactions were performed using the nanofluidic qPCR BioMark^TM^ HD system, with 48.48 and 96.96 Dynamic Array Integrated Fluidic Circuits (ICFs) (Fluidigm Corporation) following Urra et al. (27). A total of 96 validated primer sets (28) were used, including 85 primer sets targeting ARGs conferring resistance against all major classes of antibiotics [10 aminoglycosides, 14 β-lactamases, 5 FCA (fluoroquinolone, quinolone, florfenicol, chloramphenicol, and amphenicol), 13 MLSB (macrolide, lincosamide, streptogramin B), 5 multidrugs (i.e., those conferring resistance to more than one antibiotic), 4 sulfonamides, 24 tetracyclines, and 10 vancomycines], 10 primers sets targeting MGE-genes (8 genes encoding transposases and 2 genes encoding integrases), and the 16S rRNA as reference gene. DNA samples were pre-amplified with a pool of primers (final concentration for each primer pair = 50 nM; 16 PCR cycles) and then treated with exonuclease I. Subsequently, 1:10 dilutions of specific target amplification reactions were loaded onto the Dynamic Array IFCs, following the Fluidigm's Fast Gene Expression Analysis—EvaGreen® Protocol. The SsoFast^TM^ EvaGreen® Supermix with Low ROX (Bio-Rad Laboratories, Redmond, WA) was used for amplification (with a final primer concentration, both forward and reverse, of 500 nM). The cycling program consisted of 1 min at 95°C, followed by 30 cycles at 95°C for 5 s and 60°C for 20 s, followed by a melting curve. Four replicates were included for each sample. Measurements were conducted in the Gene Expression Unit of The Genomics Facility of SGIker—University of the Basque Country, Spain. Raw data obtained from the analysis were processed with the Fluidigm Real-Time PCR Analysis Software (v.3.1.3) with linear baseline correction and manual threshold settings. A threshold cycle, C_T_ value, of 31 was chosen since the highest C_T_ value obtained in our study was 30.53. A detection of an ARG or MGE-gene was considered positive when 3 out of the 4 technical replicates for each sample were above the detection limit. The value of the detection limit was used for non-amplified genes. Furthermore, a comparative C_T_ method was used to calculate ARG and MGE-gene relative abundances, normalized to the abundance of the 16S rRNA control gene, expressed as fold-change (FC) (29):

$$\begin{array}{l} \;\Delta C_{T}=C_{T\left(target\;gene\right)}-C_{T\left(16S\;rRNA\;gene\right)}\\ \Delta \Delta C_{T}=\;\Delta C_{T\left(amended\;sample\right)}-\Delta C_{T\left(unamended\;sample\right)}\\ \;FC=2^{{-\Delta \Delta C}_{T}} \end{array}$$

Real-time PCR measurements of the abundance of the 16S rRNA gene were performed to estimate total bacterial biomass, following the reaction mixtures and PCR conditions described in Epelde et al. (30). The relative copy number (GR) was calculated as the proportion of the abundance of the ARG or MGE-gene to the abundance of the 16S rRNA gene (31). Absolute ARG and MGE-gene abundances (GA_ARG, MGE_) were calculated as follows (32):

$$\begin{array}{l} \;GR=10^{\frac{\left(31-C_{T}\right)}{\left(10/3\right)}}\\ GA_{ARG,MGE}=\frac{GA_{16S}\times GR_{ARG,MGE}}{GR_{16S}} \end{array}$$

In order to assess the impact of amendments on soil prokaryotic community composition, the preparation of amplicon libraries was carried out using a dual indexing approach with sequence-specific primers (33) targeting the V4 region of the 16S rRNA gene: primers 519F (CAGCMGCCGCGGTAA) adapted from Øvreås et al. (34) and 806R (GGACTACHVGGGTWTCTAAT) from Caporaso et al. (35). Sequencing was performed with an Illumina MiSeq V2 platform and paired-end sequencing strategy (2 × 250 nt) at Tecnalia, Spain. Read paired ends were merged, quality filtered and clustered into operational taxonomic units (OTUs) as described in Lanzén et al. (33). The taxonomic classification was performed using CREST (36).

### Statistical Analysis

One-way ANOVA with Duncan's multiple-range tests was performed to compare absolute abundance values of ARGs and MGE-genes among treatments: *type of amendment* = slurry vs. fresh manure vs. aged manure, and *origin of amendment* = conventional livestock farm vs. organic livestock farm. Identical analyses were performed for crop production data. The effect of the experimental factors (*type* × *origin*) was tested by two-way ANOVA using package *agricolae* of R software (v.3.6.3). R package *vegan* (37) was used to calculate α-diversity indices (i.e., richness, Shannon's, Simpson's, Pielou's) for soil prokaryotic diversity data and 16S rRNA amplicon sequencing data visualization. Principal component analysis (PCA) of ARG and MGE-gene absolute abundances was performed using Canoco 5 (38). Venn diagram was performed to examine the overlapping, in terms of the presence of ARGs and MGE-genes, between soil and plant samples with *venn* package in R. Kendall's rank correlation coefficients, followed by Bonferroni's multiple comparisons test, between soil prokaryotic taxa (at order level) and absolute abundances of ARGs and MGE-genes (grouped by antibiotic family and MGE category) were obtained using R software.

## Results

### Amendment and Soil Physicochemical Characterization

The soil was characterized as a clay loam, with a pH of 6.2, an OM of 6.3%, a total N content of 0.32%, an Olsen P content of 3.4 mg kg^−1^ DW soil, and a K^+^ content of 395 mg kg^−1^ DW soil. Regarding the physicochemical properties of the dairy cow manure-derived amendments (Table 1), we observed that: (i) amendments from the conventional farm showed higher OM content than those from the organic farm; (ii) all pH values ranged between 8.2 and 9.4; (iii) slurry samples from both the conventional and organic farm showed higher N content, compared to fresh and aged manure; (iv) Pb, Cr, and Ni concentrations were higher in fresh and aged manure from the organic vs. the conventional farm; and (v) the following metal concentration gradient for Pb, Cr, and Ni was observed in the amendments from both the conventional and organic farm: aged manure > fresh manure > slurry.

**Table 1:** Physicochemical properties of the amendments.

	**Organic farm**			**Conventional farm**		
	**Aged manure**	**Fresh manure**	**Slurry**	**Aged manure**	**Fresh manure**	**Slurry**
Dry matter (%)	29.87	21.74	7.30	17.51	18.51	12.12
OM (%)	41.33	67.48	74.62	78.30	80.20	83.41
pH (1:25)	9.41	9.29	8.40	8.48	9.16	8.25
*N* (%)	2.78	3.45	3.97	1.94	2.32	3.38
Olsen phosphorus (g kg^−1^)	5.61	8.12	6.36	3.65	4.83	6.66
Potassium (g kg^−1^)	37.21	33.56	43.43	16.67	22.06	25.03
Cd (mg kg^−1^ DW)	0.62	0.71	0.40	0.50	0.36	0.15
Pb (mg kg^−1^ DW)	150.88	36.95	17.59	4.14	3.31	1.55
Cr (mg kg^−1^ DW)	51.80	22.32	11.58	17.58	15.23	13.54
Ni (mg kg^−1^ DW)	27.16	11.05	6.07	8.93	8.25	7.69

Concerning antibiotic concentrations in the amendments and the semi-natural grassland soil (Table 2), in the first analysis, colistin was detected in fresh and aged manure from both the conventional and organic farm. Furthermore, marbofloxacin was detected in all the amendments from the conventional farm, as well as in the slurry from the organic farm. In the second analysis carried out 2 months later, only colistin was detected in the fresh manure from the conventional farm (Table 2), indicating a possible degradation of marbofloxacin.

**Table 2:** Antibiotic concentrations in the amendments and the semi-natural grassland soil.

	**Antibiotic (μg kg^**−1**^)**	**Organic farm**			**Conventional farm**			
		**Aged manure**	**Fresh manure**	**Slurry**	**Aged manure**	**Fresh manure**	**Slurry**	**Soil**
First analysis	Colistin	470	230	<50	223	147	<50	<50
	Marbofloxacin	<5	<5	139	81	245	41.6	<5
Second analysis	Colistin	<50	<50	<50	<50	117	<50	<50

*Antibiotics whose concentration did not exceed the detection limit are not included*.

### Effect of Treatments on Crop Production

Pertaining to lettuce shoot biomass, higher values were found when the soil was amended with aged manure from both the conventional and organic farm, as well as with slurry from the conventional farm, compared to slurry from the organic farm, fresh manure from the conventional farm and the unamended control (Table 3).

**Table 3:** Effect of treatments on lettuce (shoot biomass) and wheat production (grain weight).

	**Organic farm**			**Conventional farm**			
	**Aged manure**	**Fresh manure**	**Slurry**	**Aged manure**	**Fresh manure**	**Slurry**	**Unamended control**
**LETTUCE**							
Shoot biomass (g)	138.1 ± 12.7^ab^	122.0 ± 3.9^bc^	107.4 ± 15.7^c^	142.2 ± 6.9^a^	118.3 ± 14.5^c^	142.7 ± 11.6^a^	103.8 ± 14.4^c^
**WHEAT**							
Grain weight (g)	6.4 ± 2.9^ns^	5.6 ± 1.8^ns^	6.2 ± 3.0^ns^	5.9 ± 1.6^ns^	4.3 ± 2.6^ns^	6.0 ± 1.7^ns^	4.3 ± 1.2^ns^

*Means (n = 4) and standard errors. Errors with different letters are significantly different (p < 0.05) according to Duncan‘s multiple range test. ns, non-significant*.

As far as wheat production is concerned, no statistically significant (*p* < 0.05) differences among treatments were observed. In any case, the highest value of wheat grain weight was found in pots amended with aged manure from the organic farm.

### Effect of Treatments on Biological Parameters Related to the Resistome Risk

Regarding the absolute abundances of ARGs and MGE-genes in the amendments collected from the livestock farms (Supplementary Table 1), higher values were detected for aminoglycoside resistance genes, compared to all the other genes. In turn, lower values were observed for β-lactamase, vancomycin and multidrug resistance genes. No single amendment could be identified as the best or worst amendment according to the absolute abundances of ARGs and MGE-genes (Supplementary Table 1).

**Figure 1:**
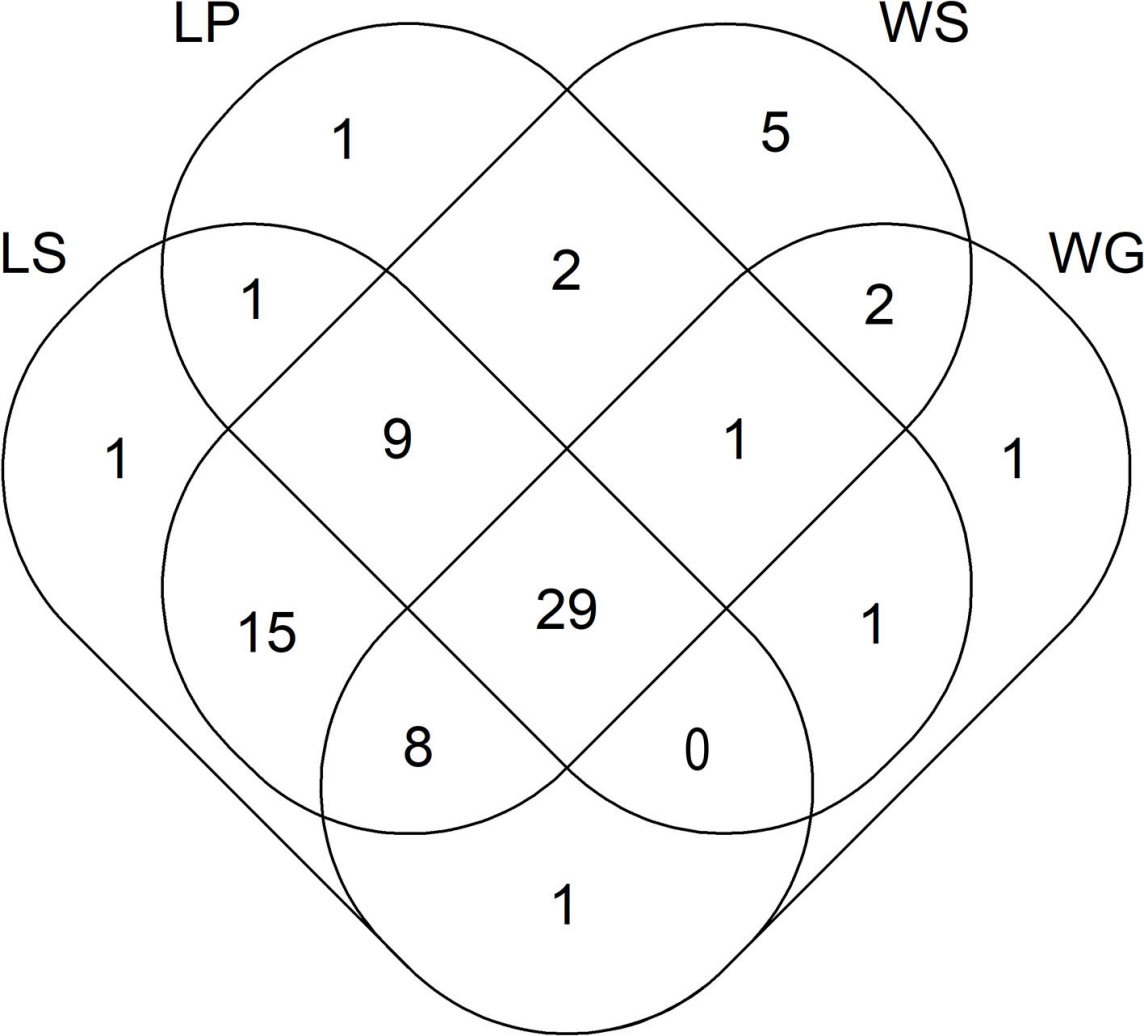
Venn diagram showing the number of ARGs and MGE-genes for lettuce and wheat samples. LS, lettuce soil; LP, lettuce plant; WS, wheat soil; WG, wheat grain.

Out of the 95 ARGs and MGE-genes quantified here, 44 and 64 genes were detected in lettuce plants and lettuce soils, respectively (Figure 1). In addition, 5 and 25 genes were exclusively detected in lettuce plants and lettuce soils, respectively (i.e., lettuce plants and lettuce soils shared 39 genes) (Figure 1). Those five genes that were only detected in lettuce plants (and not in lettuce soils) encoded resistance to β-lactamase (one gene), MLSB (one gene), vancomycin (one gene) and tetracycline (2 genes). In turn, the 25 genes that were found only in lettuce soils (and not in lettuce plants) encoded resistance to FCA (one gene), tetracycline (3 genes), multidrug (3 genes), β-lactamase (4 genes), MLSB (4 genes), aminoglycosides (5 genes), and vancomycin (5 genes).

Values of ARG absolute abundances in lettuce soils ranged from 3.25 × 10^8^ (for soil amended with slurry from the conventional farm) to 1.81 × 10^9^ (for the unamended control soil) copies g^−1^ DW soil (Supplementary Table 1). In these lettuce soils, the absolute abundance of MGE-genes was higher than that of ARGs: from 1.27 × 10^10^ (for soil amended with fresh manure from the organic farm) to 8.76 × 10^10^ (for the unamended control soil) copies g^−1^ DW soil. In lettuce soils, integrase-related genes showed the highest absolute abundance values. By contrast, multidrug resistance genes presented the lowest absolute abundance values in lettuce soils (however, differences were not statistically significant). Furthermore, the lettuce unamended (control) soil showed higher absolute abundance values for vancomycin resistance genes, compared to all the other lettuce soils. In relation to the effect of the experimental variables (type and origin of amendment) on absolute abundance values in lettuce soils, the application of aged manure led to significantly higher absolute abundances of aminoglycoside resistance genes, compared to the application of slurry (Supplementary Table 1). Moreover, lettuce soils amended with aged manure showed higher absolute abundance values for tetracycline resistance genes, compared to lettuce soils amended with fresh manure or slurry.

In lettuce plants, the absolute abundance of ARGs ranged from 1.08 × 10^8^ (for plants fertilized with slurry from the conventional farm) to 2.56 × 10^9^ (for plants fertilized with fresh manure from the conventional farm) copies g^−1^ DW plant tissue (Supplementary Table 1). The absolute abundance of MGE-genes in lettuce plants ranged from 3.53 × 10^8^ (for plants fertilized with slurry from the conventional farm) to 5.59 × 10^9^ (for plants fertilized with fresh manure from the conventional farm) copies g^−1^ DW plant tissue. In lettuce plants, the absolute abundance of ARGs was higher in plants fertilized with fresh manure from the conventional farm, compared to all the other lettuce plants, except for the unamended control (Supplementary Table 1). Genes encoding resistance to β-lactamase, FCA, multidrug, tetracycline, and vancomycin showed lower absolute abundance values than genes encoding sulfonamide and transposase in lettuce plants. Lettuce plants fertilized with fresh manure from the conventional farm showed higher absolute abundance values for aminoglycoside resistance, tetracycline resistance and transposase-related genes than lettuce plants from the other treatments (except for aged manure from the conventional farm and the unamended control). Similarly, lettuce plants fertilized with amendments from the conventional farm exhibited higher absolute abundance values of tetracycline resistance and transposase related genes than those fertilized with amendments from the organic farm.

Regarding wheat, out of the 95 ARGs and MGE-genes quantified here, 43 and 71 genes were detected in wheat grains and wheat soils, respectively (Figure 1). In addition, 3 and 31 genes were exclusively detected in wheat grains and wheat soils, respectively (i.e., wheat grains and wheat soils shared 40 genes) (Figure 1). Specifically, three tetracycline-resistance genes were only detected in wheat grain (and not in wheat soil). In turn, the 31 genes that were found only in wheat soil (and not in wheat grain) encoded resistance to multidrug (2 genes), aminoglycosides (3 genes), MLSB (3 genes), vancomycin (7 genes), β-lactamase (8 genes), and tetracycline (8 genes). The absolute abundance of ARGs in wheat soils ranged from 1.50 × 10^10^ (for wheat soil amended with slurry from the conventional farm) to 7.64 × 10^10^ (for wheat soil amended with fresh manure from the organic farm) copies g^−1^ DW soil (Supplementary Table 1). In these wheat soils, the absolute abundance of MGE-genes ranged between 3.03 × 10^11^ (for wheat soils amended with slurry from the conventional farm) to 1.17 × 10^12^ (for wheat soils amended with fresh manure from the organic farm) copies g^−1^ DW soil. On the other hand, the absolute abundance of ARGs in wheat grains ranged from 4.04 × 10^9^ (for wheat fertilized with fresh manure from the conventional farm) to 1.47 × 10^10^ (for wheat fertilized with slurry from the organic farm) copies g^−1^ DW grain. The absolute abundance of MGE-genes in wheat grains ranged from 8.74 × 10^10^ (for wheat fertilized with aged manure from the organic farm) to 2.33 × 10^11^ (for control unamended pots) copies g^−1^ DW grain. Wheat soils amended with fresh manure from both livestock farms showed higher absolute abundance values of aminoglycoside resistance genes, compared to wheat soils amended with slurry (Supplementary Table 1). Likewise, higher absolute abundance values of aminoglycoside, MLSB and vancomycin resistance genes were detected in wheat soils supplemented with amendments from the organic vs. conventional farm. Wheat grains grown with amendments from the organic farm exhibited higher absolute abundance values of FCA resistance genes, compared to those from pots treated with amendments from the conventional farm.

**Figure 2:**
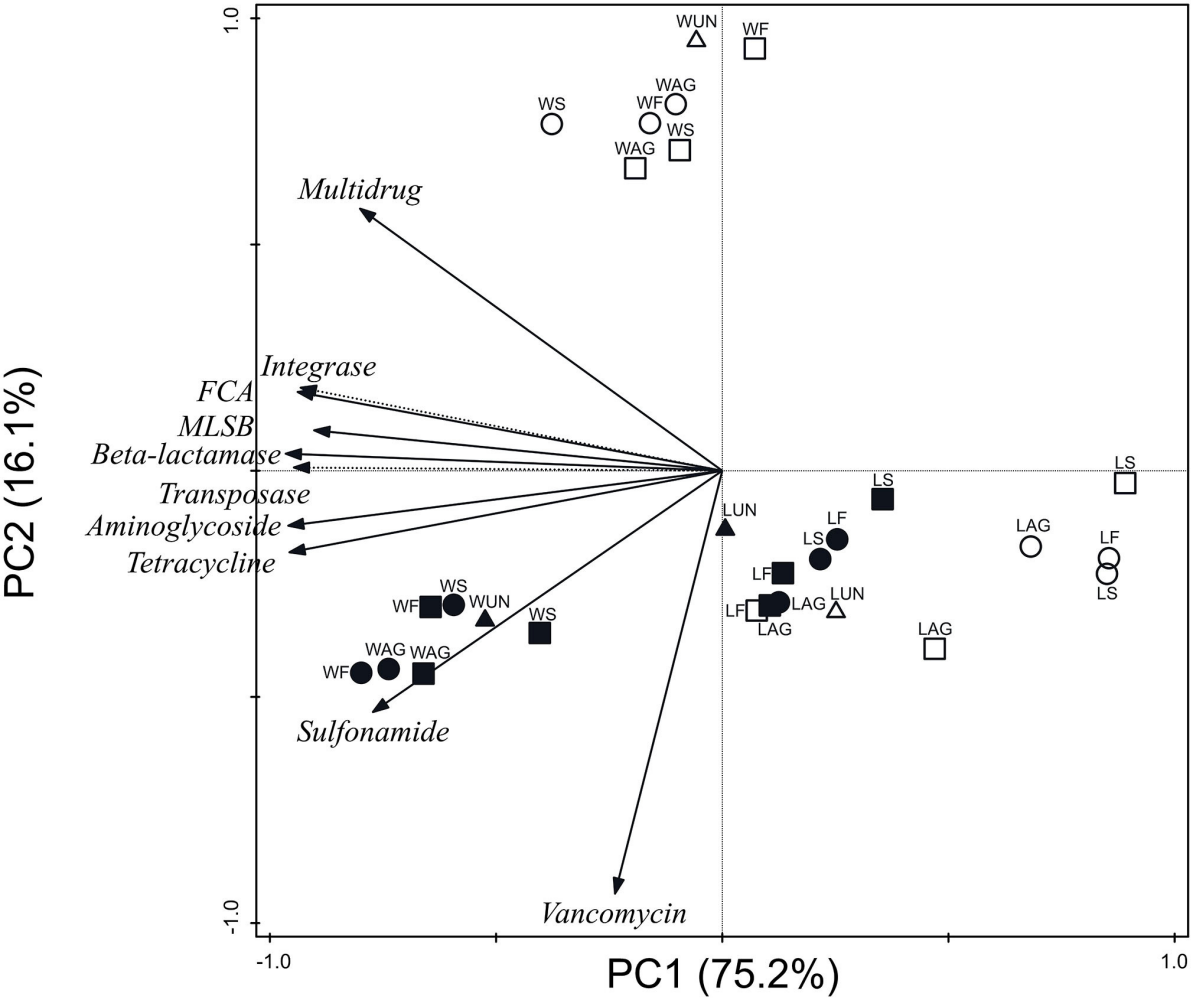
Principal component analysis of absolute abundances of ARGs and MGE-genes grouped by antibiotic family or MGE category. W, wheat samples; L, lettuce samples; AG, aged manure; F, fresh manure; S, slurry; UN, unamended. Circle: organic farm. Square: conventional farm. Empty symbol: plant. Full symbol: soil.

Figure 2 represents ARG and MGE-gene absolute abundances grouped by antibiotic family and MGE category for all soil and plant samples. The PCA clearly separated three clusters: (i) wheat soils; (ii) wheat grains; and (iii) lettuce soils and plants. The first axis (PC1) accounted for 75.2% of the total variance and showed negative loadings for the following absolute abundances: aminoglycoside, β-lactamase, FCA, integrase, MLSB, sulfonamide, tetracycline, and transposase genes. In addition, PC2 accounted for 16.1% of the total variance and showed positive loading for multidrug and negative loading for vancomycin genes.

No statistically significant differences were found among treatments for both lettuce and wheat data (soil and plant data) in relation to the relative abundances of ARGs and MGE-genes grouped by antibiotic family and MGE category (Supplementary Figures 1, 2).

Regarding the impact of treatments on soil prokaryotic diversity in lettuce soils, as reflected by Illumina MiSeq sequencing data, 73.1, 53.4, and 22.0% of the reads were taxonomically classified to order, family and genus rank, respectively. Concerning wheat soils, 67.6, 51.1, and 20.4% of the reads were classified to order, family and genus rank, respectively. Statistically significant differences were found in 15 and 3 orders in lettuce and wheat soils, respectively (Supplementary Table 2). For lettuce soils, out of these 15 orders, the following belong to the 30 most abundant orders detected in those soils: *Cytophagales, SC-I-84, Pseudonocardiales, Solirubrobacterales, C0119, KD4-96*, and *Nitrososphaerales* (Supplementary Figure 3). Similarly, out of the abovementioned three orders in wheat soils, the following two belong to the 30 most abundant orders: *Rhodospirillales* and *Desulfurellales* (Supplementary Figure 4).

Data on the impact of treatments on soil prokaryotic α-diversity are shown in Table 4. Lettuce soils amended with slurry from the conventional farm showed higher richness than those amended with slurry from the organic farm (and also higher richness, compared to the untreated control soil). Moreover, Shannon's diversity was lower in soils amended with aged manure for the organic farm and the unamended control soil, compared to all the other soils. In wheat soils, higher richness values were observed in soil amended with aged manure from the conventional farm, compared to soil amended with fresh manure and slurry from the organic farm (Table 4).

**Table 4:** Effect of treatments on soil prokaryotic diversity.

**Lettuce soil**	**Richness**	**Shannon's**	**Simpson's**	**Pielou's**
ORG_AG	3626 ± 200^cd^	6.78 ± 0.04^b^	0.997 ± 5.2E-04^ns^	0.811 ± 0.015^ns^
ORG_FRES	3894 ± 48^ab^	7.03 ± 0.04^a^	0.998 ± 2.9E-04^ns^	0.815 ± 0.005^ns^
ORG_SLU	3722 ± 65^bc^	6.99 ± 0.03^a^	0.998 ± 5.2E-05^ns^	0.814 ± 0.003^ns^
CONV_AG	3774 ± 73^abc^	6.98 ± 0.02^a^	0.998 ± 9.2E-05^ns^	0.825 ± 0.015^ns^
CONV_FRES	3784 ± 33^abc^	7.00 ± 0.03^a^	0.998 ± 1.4E-04^ns^	0.813 ± 0.009^ns^
CONV_SLU	3941 ± 149^a^	7.05 ± 0.06^a^	0.998 ± 2.9E-04^ns^	0.815 ± 0.007^ns^
UNAMEN	3550 ± 95^d^	6.89 ± 0.04^bc^	0.997 ± 1.1E-04^ns^	0.808 ± 0.002^ns^
**Wheat soil**	**Richness**	**Shannon's**	**Simpson's**	**Pielou's**
ORG_AG	4517 ± 85^ab^	7.04 ± 0.04^ns^	0.998 ± 2.0E-04^ns^	0.817 ± 0.010^ns^
ORG_FRES	4285 ± 176^b^	6.95 ± 0.07^ns^	0.997 ± 3.1E-04^ns^	0.810 ± 0.005^ns^
ORG_SLU	4268 ± 259^b^	6.91 ± 0.12^ns^	0.997 ± 3.6E-04^ns^	0.811 ± 0.014^ns^
CONV_AG	4710 ± 53^a^	7.08 ± 0.03^ns^	0.998 ± 1.8E-04^ns^	0.817 ± 0.005^ns^
CONV_FRES	4530 ± 98^ab^	7.00 ± 0.04^ns^	0.997 ± 2.1E-04^ns^	0.818 ± 0.010^ns^
CONV_SLU	4510 ± 289^ab^	7.01 ± 0.14^ns^	0.998 ± 4.3E-04^ns^	0.814 ± 0.010^ns^
UNAMEN	4441 ± 47^ab^	6.95 ± 0.04^ns^	0.997 ± 3.0E-04^ns^	0.805 ± 0.008^ns^

*Means (n = 4) and standard errors. Errors with different letters are significantly different (p < 0.05) according to Duncan‘s multiple range test. ORG_AG, aged manure form organic farm; ORG_FRES, fresh manure from organic farm; ORG_SLU, slurry from organic farm; CONV_AG, aged manure from conventional farm; CONV_FRES, fresh manure from conventional farm; CONV_SLU, slurry from conventional farm; UNAMEN, unamended control. ns, non-significant*.

In lettuce soils, Kendall's rank correlation coefficients showed significant correlations (positive and negative) among 43 orders and 7 ARG and 2 MGE-gene absolute abundances grouped by antibiotic family and MGE category (Supplementary Table 2). Among these 43 orders, the following five presented multiresistance: *Micrococcales, Pseudonocardiales, Rhizobiales, Rubrobacterales*, and *Solirubrobacterales* (Supplementary Table 2). The orders *Micrococcales, Pseudonocardiales, Rhizobiales*, and *Solirubrobacterales* appeared in the list of the 30 most abundant orders in lettuce soils (Supplementary Figure 3). The order *Pseudonocardiales* was positively correlated with genes encoding resistance to MLSB, tetracycline and vancomycin (Supplementary Table 2). The lettuce unamended soil showed higher abundance of *Pseudonocardiales* than the other soils (Supplementary Table 3). Fifteen orders showed, at least, two negative correlations with ARG and MGE-gene absolute abundances (Supplementary Table 2).

In wheat soils, Kendall's rank correlation coefficients showed significant correlations (positive and negative) among 14 orders and 6 ARG and 2 MGE-gene absolute abundances grouped by antibiotic family and MGE category (Supplementary Table 2). Among these 14 orders, the following three presented multiresistance: *Limnochordales, Tepidisphaerales*, and *WN-HWB-116* (Supplementary Table 2).

As far as differences between lettuce and wheat pots, wheat soil and grain samples showed higher absolute abundances of ARGs and MGE-genes than lettuce soil and plant samples (Supplementary Table 4). In terms of absolute abundances, the highest number of statistically significant differences between lettuce and wheat soils was observed in soils amended with fresh manure from the organic farm.

## Discussion

The incorporation of organic amendments into agricultural soil as fertilizers often increases soil OM content (39) and fertility, and results in an overall improvement of soil quality (8). In particular, organic farming practices promote the maintenance and enhancement of soil OM and fertility by means of the application of farmyard manure and similar organic amendments. In Europe, the area under organic farming increased from 10.0 million hectares in 2012 to 13.4 million hectares in 2018 (Eurostat Statistics for Organic Farming). Despite the abovementioned well-recognized benefits, there is increasing concern about the use of manure-derived amendments as organic fertilizers since their application entails a variety of environmental risks such as, for instance, the emergence, maintenance and dissemination of AR in agricultural soils and crops (6, 17, 40). The application of manure-derived amendments to agricultural soil can also lead to pronounced changes in the diversity and composition of soil microbial communities (41), with potential concomitant alterations of soil functioning. We hypothesized that the resistome risk would be higher in soils and plants amended with animal wastes from conventional livestock farming vs. organic livestock farming (after all, the administration of antibiotics to animals raised under organic farming is limited by regulations). Nonetheless, such hypothesis is not supported by the results of our study. Actually, even regarding the concentration of antibiotics in the amendments collected from the organic vs. conventional farm, no clear differences were observed, which could be due to the fact that organic farms do apply antibiotics in some specific cases, e.g., during a long-term mastitis.

As described above, a large proportion (30–90%) of the antibiotics administered to livestock are not fully metabolized and are then excreted, together with their transformation products, into the environment along with the feces and urine (5). The amount and rate of antibiotic excretion varies greatly among animal species and age (42, 43), type and dosage of antibiotic, form of administration, etc. (44). As an example, the following concentrations (mg kg^−1^) have been reported for dairy cow manure: 0.43–2.69 for tetracycline, 0.21–10.37 for oxytetracycline, 0.61–1.94 for chlortetracycline, 0.22–1.02 for sulfamethoxazole, 0.43–1.76 for norfloxacin and 0.46–4.17 for enrofloxacin (45, 46). On the other hand, once introduced into the soil matrix, antibiotics are susceptible to a variety of processes, such as adsorption, microbial transformation, photodegradation, plant uptake, sequestration, transport (leaching, runoff), etc. (13, 42, 47, 48). In contrast with other studies (49–51), macrolides, sulphonamides, and tetracyclines were not detected in any of the amendments studied here. Actually, in the first analysis, out of the 57 antibiotics analyzed here, only colistin and marbofloxacin were detected. In the second analysis, only colistin (117 μg kg^−1^) was detected in one of the amendments, i.e., fresh manure from the conventional farm. Nonetheless, we did find genes encoding resistance to those antibiotics in the amendments, which could be due to the fact that: (i) the antibiotics were already completely degraded but the ARGs persisted in the amendments despite the absence of the antibiotics; (ii) antibiotic transformation products, still capable of bioactive effect, are responsible for the induction of the emergence of ARGs in the amendments (42); and/or (iii) although antibiotic concentrations in the amendments were below the detection limit of the technique, sub-inhibitory concentrations result in an enough level of selective pressure to induce AR (52). Furthermore, antibiotic sub-inhibitory concentrations are known to induce horizontal gene transfer (53), which could spread ARGs among different bacterial populations. Interestingly, some studies (40, 54) have reported an increase in AR in soils amended with manure from animals that had not been subjected to any antibiotic treatment.

In our study, the amendment that showed the highest absolute abundances of ARGs and MGE-genes was the slurry from the conventional farm, but this highest resistome risk was then not reflected, as one would expect, in those soils and crops amended with such slurry. Actually, despite the fact that the slurry from both livestock farms presented greater values of absolute abundance for transposase, aminoglycoside, MLSB, tetracycline and multidrug resistance genes (compared to the other amendments), lettuce soils amended with such slurry showed a lower resistome risk than when fertilized with the other amendments. Also, the absolute abundances for aminoglycoside resistance genes were lower in wheat soils amended with slurry vs. fresh and aged manure. Remarkably, within the same treatment, the resistome risk differed between the amendment, the amended soil and, finally, the crop. In other words, according to our data, the resistome risk in manure-amended crops cannot be directly inferred from the analysis of the amendments themselves. Although aging and composting are both effective processes (composting is certainly more effective than aging in this respect) for reducing the concentration of antibiotics and the total abundance of ARGs, the trend in some ARGs is highly gene-specific (55). In our case, the manure was not composted following a controlled procedure, but simply aged for approximately 6 months. In any case, dairy fresh manure from both the conventional and the organic farm presented higher absolute abundances of *intI1, sul2*, and 7 tetracycline-resistance than those reported in previous studies (56–58).

On the other hand, slurry samples from both livestock farms showed the lowest metal concentrations, compared to aged and fresh manure. The *co-selection* of antibiotic and metal resistance in bacteria, due to *co-resistance* (when two or more different resistance genes are located on the same genetic element, e.g., a plasmid or a transposon) or *cross-resistance* (when a single mechanism confers resistance to both antibiotics and metals, e.g., an efflux pump) mechanisms, is widely known (59–62). Moreover, *co-regulatory mechanisms* (when genes that confer resistance to different compounds are controlled by a single regulatory gene) can promote antibiotic-metal co-selection processes. In this respect, Perron et al. (46) reported that the regulatory protein CzcR regulates (i) the expression of the CzcCBA efflux pump, which confers resistance to Zn, Cd and Co; and (ii) the expression of the OprD porin, the route of entry of carbapenems in bacteria. This co-selection phenomenon is of the utmost importance as it can be responsible for the maintenance and dissemination of AR in the absence of antibiotics. In our study, as abovementioned, the values of ARG and MGE-gene absolute abundances were lower in aged and fresh manure than in slurry, but it is possible that the higher metal concentrations detected in aged and fresh manure (vs. slurry) could induce the spread of ARGs once applied to the agricultural soil.

Overall, the values of absolute abundance of ARGs and MGE-genes were higher in soil vs. plant samples, in agreement with previous studies (63, 64). Soils are important reservoir of ARGs (16, 65). In any event, the typology of ARGs found in lettuce and wheat grains was robustly dependent on the typology of ARGs observed in the corresponding soil. Relevantly, much higher absolute abundances of MGE-genes vs. ARGs were detected in both soil and plant samples, pointing out to a high potential risk of dissemination of AR in the studied soils and crops. In addition to the physical contact and interactions among the plants, the soil and the amendments, in some cases, the water used for irrigation is another factor to be considered, as it might be contaminated with ARGs (66). However, in our study, this is not a relevant factor since the same tap water was used to irrigate all the treatments.

Furthermore, we found higher ARG and MGE-gene absolute abundances in wheat vs. lettuce soils. Plants are known to regulate rhizosphere microbial communities through the excretion of root exudates (67). The composition and quantity of root exudates greatly vary depending on the specific plant species and its physiological status (68, 69). The type of crop (lettuce vs. wheat), dose of amendment (here adjusted to 100 vs. 180 kg N ha^−1^ for lettuce and wheat plants, respectively), duration of plant growth until harvest (44 vs. 171 days for lettuce and wheat plants, respectively), type of root system (pivotant vs. fasciculate for lettuce and wheat plants, respectively), and the amount and composition of the rhizodeposition are all factors that can affect the composition of soil microbial communities and the fate and distribution of ARGs and MGE-genes in agricultural soils. No significant differences were observed, in terms of the absolute abundances of ARGs and MGE-genes, between unamended lettuce soils and unamended wheat soils (neither between lettuce and wheat grain samples), which indicates that the amendment application was responsible for the observed differences among treatments.

Although Zhang et al. (64) observed higher ARG abundances in manure-amended lettuce soils (the abundance of ARGs ranged from 4.37 × 10^9^ to 2.02 × 10^10^ in soils), compared to ours, the transfer of those ARGs from the lettuce soil to the lettuce was approximately between one and two orders of magnitude higher in our study (the abundance of ARGs ranged from 7.45 × 10^6^ to 8.24 × 10^7^ in plant samples). In lettuce soils, the unamended control showed the highest abundance of vancomycin resistance genes. Antibiotic resistance genes have not only been found in antibiotic-free soil (70) but also in environments (e.g., permafrost, isolated caves) that have remained isolated from the impact of anthropic activity much before the beginning of the use of antibiotics for the preventive and curative treatment of bacterial infectious diseases in medicine and veterinary (71, 72).

Regarding the possible links between the presence of certain prokaryotic taxa and AR profiles, the order *Pseudonocardiales* presented a positive correlation with vancomycin resistance genes. Several strains belonging to *Pseudonocardiales* are known to produce biologically active products, such as erythromycin, rifamycin, and vancomycin (73). The unamended control soil showed significantly higher abundance of *Pseudonocardiales* than the other treated soils (and, as already mentioned, the unamended lettuce soil showed the highest abundance of vancomycin resistance genes). In general, the unamended lettuce soil showed lower abundances of those orders negatively correlated with vancomycin resistance genes (*Cytophagales, Obscuribacterales*, and *SAR324*), compared to the other treated soils. Many authors (74, 75) have reported that changes in the composition of prokaryotic communities appear to be the key drivers for the magnitude and profile of the antibiotic resistome. The values of bacterial richness and Shannon's diversity detected in the unamended control soil were significantly lower than those observed in the other soils (except for soils amended with aged manure from the organic farm). These data suggest that vancomycin resistance genes most likely did not enter the soil matrix through the application of the amendments, but that they existed previously in such soil. Chaudhry et al. (76) found that the application of amendments to soil could lead to an increase of (i) overall bacterial diversity; and (ii) the dominance of certain bacterial taxa which could then play important roles in a variety of soil processes. Highly diverse soil microbial communities can, for instance, act as a biological barrier against biological invasion (77). The decline in microbial diversity has often been related to a loss of ecosystem multi-functionality (78).

## Conclusions

Despite our initial hypotheses, no single treatment could be identified as the best or worst treatment regarding the risk of antibiotic resistance in soil and plant samples. Interestingly, within the same treatment, the resistome risk differed between the amendment, the amended soil and, finally, the crop. In other words, according to our data, the resistome risk in manure-amended crops cannot be directly inferred from the analysis of the amendments themselves. Then, we concluded that, depending on the specific question under study, the analysis of the resistome risk should specifically focus on the amendment, the amended soil or the crop. In any case, our results confirm the risk of AR dissemination in agricultural settings where dairy cow manure-derived amendments are used as fertilizers. In this respect, much higher absolute abundances of MGE-genes vs. ARGs were detected in both soil and plant samples, pointing out to a high potential risk of dissemination of AR in the studied soils and crops.

## Data Availability Statement

The dataset presented in the study are publicly available. This data can be found here: https://www.ebi.ac.uk/ena, PRJEB41541.

## Author Contributions

LE, IA, and CG designed the study. LJ and LE performed the analytical work and data treatment. LJ, LE, IA, and CG wrote the manuscript. All authors revised the final version of the manuscript.

## Conflict of Interest

The authors declare that the research was conducted in the absence of any commercial or financial relationships that could be construed as a potential conflict of interest.
