# Supplementary material for: Antibiotic Resistance in Agricultural Soil and Crops Associated to the Application of Cow Manure-Derived Amendments From Conventional and Organic Livestock Farms
Source: Front Vet Sci. 2021 Feb 23;8:633858. doi: 10.3389/fvets.2021.633858 (PMC7940349; doi:10.3389/fvets.2021.633858)
Supplement: Supplementary file 1 [file Data_Sheet_1.docx]

Supplementary Material

# Supplementary Figures and Tables

**Supplementary Figure 1.** Relative abundance of ARGs and MGE-genes (grouped by antibiotic family or MGE category) for lettuce soil and plant samples. Data are expressed as fold-change and plotted on a log scale. ORG_AG: aged manure from organic farm; ORG_FRES: fresh manure from organic farm; ORG_SLU: slurry from organic farm; CONV_AG: aged manure from conventional farm; CONV_FRES: fresh manure from conventional farm; CONV_SLU: slurry from conventional farm; UNAM: unamended.

**Supplementary Figure 2.** Relative abundances of ARGs and MGE-genes (grouped by antibiotic family or MGE category) for wheat soil and plant (grain) samples. Data are expressed as fold-change and plotted on a log scale. ORG_AG: aged manure from organic farm; ORG_FRES: fresh manure from organic farm; ORG_SLU: slurry from organic farm; CONV_AG: aged manure from conventional farm; CONV_FRES: fresh manure from conventional farm; CONV_SLU: slurry from conventional farm; UNAM: unamended.


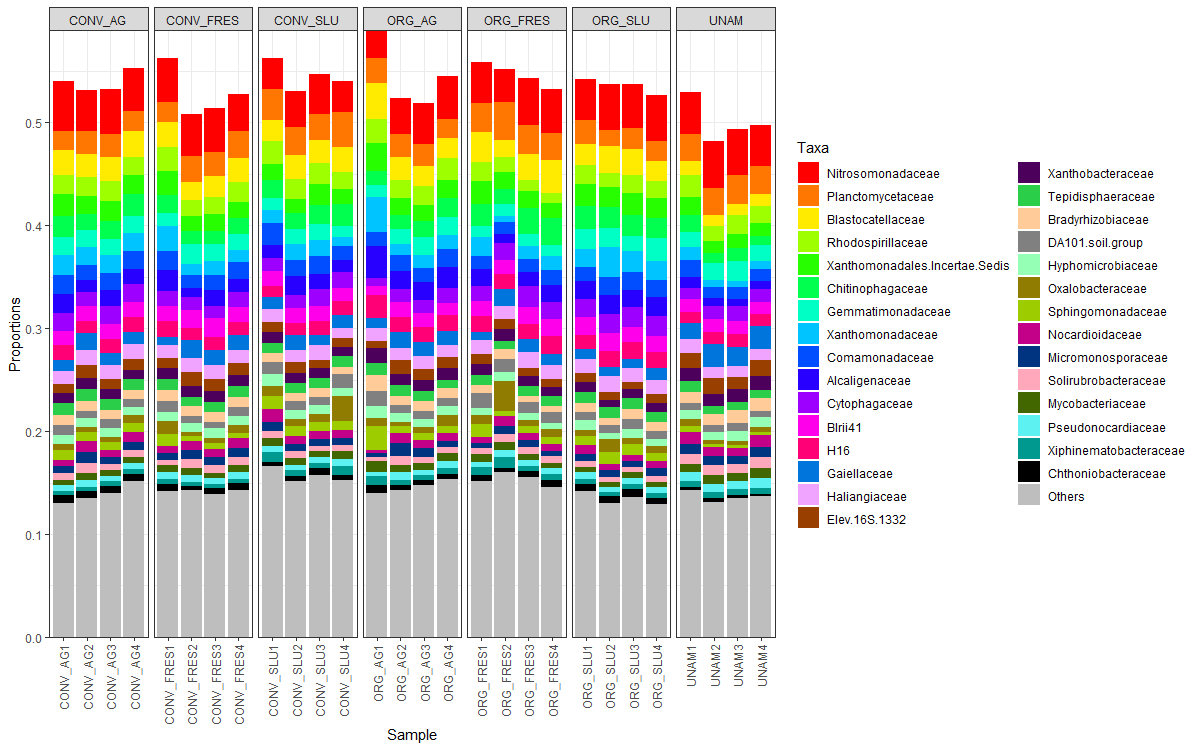
**Supplementary Figure 3.** The 30 most abundant bacterial orders in lettuce soils. ORG_AG: aged manure from organic farm; ORG_FRES: fresh manure from organic farm; ORG_SLU: slurry from organic farm; CONV_AG: aged manure from conventional farm; CONV_FRES: fresh manure from conventional farm; CONV_SLU: slurry from conventional farm; UNAM: unamended.


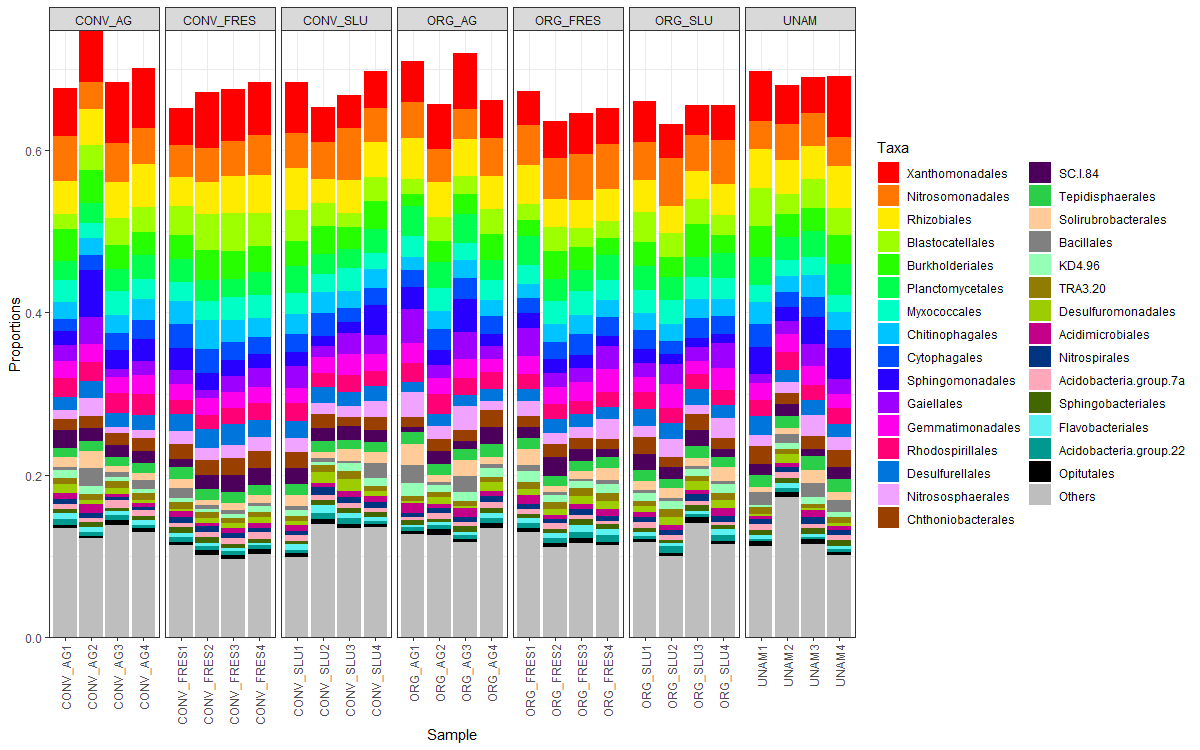


**Supplementary Figure 4.** The 30 most abundant bacterial orders in wheat soils. ORG_AG: aged manure from organic farm; ORG_FRES: fresh manure from organic farm; ORG_SLU: slurry from organic farm; CONV_AG: aged manure from conventional farm; CONV_FRES: fresh manure from conventional farm; CONV_SLU: slurry from conventional farm; UNAM: unamended.

**Supplementary Table 1.** Absolute abundances of ARGs and MGE-genes in the amendments, lettuce soils, lettuce plants, wheat soils and wheat (grain) plants. Different letters indicate significant (p<0.05) differences according to one-way and two-way ANOVA and Duncan`s multiple range test. ORG_AG: aged manure from organic farm; ORG_FRES: fresh manure from organic farm; ORG_SLU: slurry from organic farm; CONV_AG: aged manure from conventional farm; CONV_FRES: fresh manure from conventional farm; CONV_SLU: slurry from conventional farm; UNAMEN: unamended.TYPE: aged manure *vs.* fresh manure *vs*. slurry. ORIGIN: organic *vs.* conventional. *: p<0.05; **: p<0.01; ***: p<0.001; ns: non-significant.

| **AMENDMENT** | **Integrase** | **Transposase** | **Aminoglycoside** | **β-lactamase** | **FCA** | **MLSB** | **Sulfonamide** | **Tetracycline** | **Vancomycin** | **Multidrug** | **Total MGE (average)** | **Total ARG (average)** |
| --- | --- | --- | --- | --- | --- | --- | --- | --- | --- | --- | --- | --- |
| ORG_AG | 2.44E+10^b^ | 1.49E+11^b^ | 1.36E+12^bc^ | 8.37E+08^ns^ | 2.36E+10^ns^ | 2.91E+10^b^ | 5.11E+11^ns^ | 5.88E+11^abc^ | 4.15E+08^ns^ | 1.43E+08^b^ | 8.68E+10^ns^ | 3.14E+11^ns^ |
| ORG_FRES | 1.13E+10^b^ | 2.80E+11^b^ | 8.35E+11^bc^ | 2.29E+07^ns^ | 5.82E+09^ns^ | 1.18E+10^b^ | 4.91E+11^ns^ | 2.62E+11^bc^ | 5.80E+07^ns^ | 8.39E+06^b^ | 1.46E+11^ns^ | 2.01E+11^ns^ |
| ORG_SLU | 1.73E+10^b^ | 1.19E+12^a^ | 3.57E+12^ab^ | 4.57E+07^ns^ | 8.57E+09^ns^ | 1.07E+11^ab^ | 5.49E+11^ns^ | 8.27E+11^ab^ | 6.40E+07^ns^ | 9.83E+07^b^ | 6.04E+11^ns^ | 6.33E+11^ns^ |
| CONV_AG | 9.14E+09^b^ | 1.66E+11^b^ | 5.11E+11^c^ | 1.42E+08^ns^ | 2.28E+10^ns^ | 4.54E+09^b^ | 1.70E+11^ns^ | 9.81E+10^c^ | 4.13E+07^ns^ | 7.87E+06^b^ | 8.74E+10^ns^ | 1.01E+11^ns^ |
| CONV_FRES | 3.42E+11^a^ | 2.85E+11^b^ | 1.86E+12^bc^ | 1.01E+08^ns^ | 3.02E+10^ns^ | 4.44E+10^b^ | 3.82E+11^ns^ | 4.55E+11^abc^ | 2.85E+07^ns^ | 1.03E+06^b^ | 3.14E+11^ns^ | 3.46E+11^ns^ |
| CONV_SLU | 1.11E+11^ab^ | 1.59E+12^a^ | 4.84E+12^a^ | 4.64E+08^ns^ | 1.07E+10^ns^ | 1.51E+11^a^ | 9.32E+11^ns^ | 9.72E+11^a^ | 2.25E+08^ns^ | 1.01E+09^a^ | 8.49E+11^ns^ | 8.63E+11^ns^ |
| TYPE | ns | *** | *** | ns | ns | ** | ns | * | ns | * | ns | ns |
| ORIGIN | ns | ns | ns | ns | ns | ns | ns | ns | ns | ns | ns | ns |
| **LETTUCE SOIL** | **Integrase** | **Transposase** | **Aminoglycoside** | **β-lactamase** | **FCA** | **MLSB** | **Sulfonamide** | **Tetracycline** | **Vancomycin** | **Multidrug** | **Total MGE (average)** | **Total ARG (average)** |
| ORG_AG | 3.10E+10^ns^ | 9.04E+09^ns^ | 5.46E+09^ns^ | 4.58E+08^ns^ | 2.53E+08^ns^ | 9.95E+08^ns^ | 3.62E+09^ns^ | 9.61E+08^ns^ | 5.23E+08^b^ | 2.41E+07^ns^ | 2.00E+10^ns^ | 1.54E+09^ns^ |
| ORG_FRES | 1.91E+10^ns^ | 6.32E+09^ns^ | 2.87E+09^ns^ | 2.66E+08^ns^ | 1.56E+08^ns^ | 4.79E+08^ns^ | 1.12E+09^ns^ | 6.52E+08^ns^ | 2.77E+08^b^ | 4.31E+07^ns^ | 1.27E+10^ns^ | 7.33E+08^ns^ |
| ORG_SLU | 3.53E+10^ns^ | 2.95E+09^ns^ | 2.69E+09^ns^ | 4.73E+08^ns^ | 2.72E+08^ns^ | 5.80E+08^ns^ | 2.20E+09^ns^ | 6.95E+08^ns^ | 4.34E+08^b^ | 4.43E+07^ns^ | 1.91E+10^ns^ | 9.24E+08^ns^ |
| CONV_AG | 4.15E+10^ns^ | 1.28E+10^ns^ | 5.14E+09^ns^ | 5.45E+08^ns^ | 2.40E+08^ns^ | 4.95E+08^ns^ | 4.30E+09^ns^ | 1.22E+09^ns^ | 6.47E+08^b^ | 3.95E+07^ns^ | 2.72E+10^ns^ | 1.58E+09^ns^ |
| CONV_FRES | 4.17E+10^ns^ | 8.07E+09^ns^ | 4.40E+09^ns^ | 5.19E+08^ns^ | 3.50E+08^ns^ | 1.04E+09^ns^ | 1.97E+09^ns^ | 8.85E+08^ns^ | 7.61E+08^b^ | 2.64E+07^ns^ | 2.49E+10^ns^ | 1.24E+09^ns^ |
| CONV_SLU | 1.76E+10^ns^ | 1.11E+10^ns^ | 5.69E+08^ns^ | 2.40E+08^ns^ | 6.96E+07^ns^ | 3.67E+08^ns^ | 3.24E+08^ns^ | 6.84E+08^ns^ | 3.19E+08^b^ | 2.80E+07^ns^ | 1.43E+10^ns^ | 3.25E+08^ns^ |
| UNAMEN | 1.65E+11^ns^ | 1.01E+10^ns^ | 1.63E+09^ns^ | 2.59E+09^ns^ | 4.33E+08^ns^ | 2.93E+09^ns^ | 1.44E+09^ns^ | 2.37E+09^ns^ | 2.99E+09^a^ | 1.12E+08^ns^ | 8.76E+10^ns^ | 1.81E+09^ns^ |
| TYPE | ns | ns | * | ns | ns | ns | ns | * | ns | ns | ns | ns |
| ORIGIN | ns | ns | ns | ns | ns | ns | ns | ns | ns | ns | ns | ns |
| TYPE X ORIGIN | ns | ns | ns | ns | * | ns | ns | ns | ns | ns | ns | ns |
| **LETTUCE PLANT** | **Integrase** | **Transposase** | **Aminoglycoside** | **β-lactamase** | **FCA** | **MLSB** | **Sulfonamide** | **Tetracycline** | **Vancomycin** | **Multidrug** | **Total MGE (average)** | **Total ARG (average)** |
| ORG_AG | 1.89E+09^ns^ | 5.82E+08^b^ | 9.43E+08^b^ | 1.18E+08^ns^ | 1.59E+07^ns^ | 1.04E+08^ns^ | 8.81E+08^ns^ | 8.51E+07^b^ | 1.15E+06^ns^ | 1.15E+06^ns^ | 1.24E+09^ns^ | 2.69E+08^b^ |
| ORG_FRES | 4.02E+08^ns^ | 4.61E+08^b^ | 5.17E+08^b^ | 5.44E+07^ns^ | 8.17E+06^ns^ | 4.42E+07^ns^ | 6.68E+08^ns^ | 4.27E+07^b^ | 2.94E+05^ns^ | 2.94E+05^ns^ | 4.31E+08^ns^ | 1.67E+08^b^ |
| ORG_SLU | 2.64E+08^ns^ | 5.30E+08^b^ | 4.34E+08^b^ | 7.02E+07^ns^ | 1.03E+07^ns^ | 2.13E+07^ns^ | 9.20E+08^ns^ | 5.25E+07^b^ | 4.47E+05^ns^ | 4.47E+05^ns^ | 3.97E+08^ns^ | 1.89E+08^b^ |
| CONV_AG | 7.17E+08^ns^ | 2.89E+09^ab^ | 1.26E+09^b^ | 1.42E+08^ns^ | 4.71E+06^ns^ | 3.97E+09^ns^ | 1.90E+09^ns^ | 5.46E+08^ab^ | 3.38E+07^ns^ | 1.14E+06^ns^ | 1.80E+09^ns^ | 9.38E+08^ab^ |
| CONV_FRES | 2.53E+09^ns^ | 8.65E+09^a^ | 7.13E+09^a^ | 9.28E+08^ns^ | 2.88E+08^ns^ | 1.65E+09^ns^ | 8.66E+09^ns^ | 1.05E+09^a^ | 5.75E+08^ns^ | 2.08E+08^ns^ | 5.59E+09^ns^ | 2.56E+09^a^ |
| CONV_SLU | 4.23E+08^ns^ | 2.83E+08^b^ | 2.75E+08^b^ | 3.04E+07^ns^ | 2.43E+07^ns^ | 3.58E+07^ns^ | 4.70E+08^ns^ | 2.40E+07^b^ | 2.14E+05^ns^ | 1.59E+06^ns^ | 3.53E+08^ns^ | 1.08E+08^b^ |
| UNAMEN | 1.02E+09^ns^ | 5.66E+09^ab^ | 4.18E+09^ab^ | 5.51E+08^ns^ | 3.15E+08^ns^ | 4.91E+08^ns^ | 6.80E+09^ns^ | 6.41E+08^ab^ | 7.51E+06^ns^ | 7.51E+06^ns^ | 3.34E+09^ns^ | 1.62E+09^ab^ |
| TYPE | ns | ns | ns | ns | ns | ns | ns | ns | ns | ns | ns | ns |
| ORIGIN | ns | * | ns | ns | ns | ns | ns | * | ns | ns | ns | * |
| TYPE X ORIGIN | ns | ns | ns | ns | ns | ns | ns | ns | ns | ns | ns | ns |
| **WHEAT SOIL** | **Integrase** | **Transposase** | **Aminoglycoside** | **β-lactamase** | **FCA** | **MLSB** | **Sulfonamide** | **Tetracycline** | **Vancomycin** | **Multidrug** | **Total MGE (average)** | **Total ARG (average)** |
| ORG_AG | 1.95E+12^ns^ | 6.66E+10^ns^ | 1.83E+11^ns^ | 5.16E+10^ns^ | 1.40E+10^ns^ | 1.37E+10^ns^ | 1.34E+11^ns^ | 3.91E+10^ns^ | 2.29E+10^ns^ | 2.73E+09^ns^ | 1.01E+12^ns^ | 5.77E+10^ns^ |
| ORG_FRES | 2.19E+12^ns^ | 1.42E+11^ns^ | 3.27E+11^ns^ | 5.70E+10^ns^ | 1.09E+10^ns^ | 2.17E+10^ns^ | 1.19E+11^ns^ | 4.38E+10^ns^ | 2.91E+10^ns^ | 2.65E+09^ns^ | 1.17E+12^ns^ | 7.64E+10^ns^ |
| ORG_SLU | 1.62E+12^ns^ | 4.11E+10^ns^ | 8.40E+10^ns^ | 2.87E+10^ns^ | 7.42E+09^ns^ | 1.68E+10^ns^ | 1.93E+10^ns^ | 2.73E+10^ns^ | 2.72E+10^ns^ | 1.59E+09^ns^ | 8.33E+11^ns^ | 2.66E+10^ns^ |
| CONV_AG | 1.19E+12^ns^ | 6.39E+10^ns^ | 1.16E+11^ns^ | 3.37E+10^ns^ | 9.76E+09^ns^ | 8.23E+09^ns^ | 1.17E+11^ns^ | 3.50E+10^ns^ | 1.40E+10^ns^ | 1.65E+09^ns^ | 6.27E+11^ns^ | 4.19E+10^ns^ |
| CONV_FRES | 1.89E+12^ns^ | 4.62E+10^ns^ | 9.73E+10^ns^ | 3.16E+10^ns^ | 1.68E+10^ns^ | 1.32E+10^ns^ | 3.86E+10^ns^ | 2.49E+10^ns^ | 1.83E+10^ns^ | 2.66E+09^ns^ | 9.66E+11^ns^ | 3.04E+10^ns^ |
| CONV_SLU | 5.89E+11^ns^ | 1.69E+10^ns^ | 4.29E+10^ns^ | 2.01E+10^ns^ | 9.52E+08^ns^ | 5.59E+09^ns^ | 2.88E+10^ns^ | 1.29E+10^ns^ | 7.95E+09^ns^ | 1.07E+09^ns^ | 3.03E+11^ns^ | 1.50E+10^ns^ |
| UNAMEN | 1.45E+12^ns^ | 2.91E+10^ns^ | 2.99E+10^ns^ | 2.95E+10^ns^ | 2.39E+09^ns^ | 1.38E+10^ns^ | 3.69E+10^ns^ | 1.84E+10^ns^ | 3.16E+10^ns^ | 2.79E+09^ns^ | 7.42E+11^ns^ | 2.06E+10^ns^ |
| TYPE | ns | ns | * | ns | ns | ns | ns | ns | ns | ns | ns | ns |
| ORIGIN | ns | ns | * | ns | ns | * | ns | ns | * | ns | ns | ns |
| TYPE X ORIGIN | ns | ns | ns | ns | ns | ns | ns | ns | ns | ns | ns | ns |
| **WHEAT GRAIN** | **Integrase** | **Transposase** | **Aminoglycoside** | **β-lactamase** | **FCA** | **MLSB** | **Sulfonamide** | **Tetracycline** | **Vancomycin** | **Multidrug** | **Total MGE (average)** | **Total ARG (average)** |
| ORG_AG | 2.33E+11^ns^ | 8.81E+09^ns^ | 9.42E+09^ns^ | 1.50E+09^ns^ | 3.76E+09^ns^ | 8.12E+09^ns^ | 1.91E+09^ns^ | 3.15E+08^ns^ | - | 1.45E+10^ns^ | 1.21E+11^ns^ | 5.65E+09^ns^ |
| ORG_FRES | 1.66E+11^ns^ | 8.87E+09^ns^ | 5.56E+09^ns^ | 9.55E+09^ns^ | 1.58E+09^ns^ | 7.52E+09^ns^ | 1.43E+09^ns^ | 2.80E+09^ns^ | - | 1.33E+10^ns^ | 8.74E+10^ns^ | 5.95E+09^ns^ |
| ORG_SLU | 2.25E+11^ns^ | 2.68E+10^ns^ | 2.77E+10^ns^ | 8.24E+09^ns^ | 9.85E+09^ns^ | 2.63E+10^ns^ | 2.58E+09^ns^ | 4.32E+09^ns^ | - | 2.37E+10^ns^ | 1.26E+11^ns^ | 1.47E+10^ns^ |
| CONV_AG | 2.53E+11^ns^ | 1.35E+10^ns^ | 2.09E+10^ns^ | 3.72E+09^ns^ | 1.49E+09^ns^ | 2.29E+09^ns^ | 3.45E+09^ns^ | 2.64E+09^ns^ | - | 1.76E+10^ns^ | 1.33E+11^ns^ | 7.44E+09^ns^ |
| CONV_FRES | 2.04E+11^ns^ | 4.94E+09^ns^ | 3.31E+09^ns^ | 8.01E+09^ns^ | 2.02E+08^ns^ | 3.63E+09^ns^ | 2.61E+08^ns^ | 2.31E+08^ns^ | - | 1.26E+10^ns^ | 1.04E+11^ns^ | 4.04E+09^ns^ |
| CONV_SLU | 2.24E+11^ns^ | 2.37E+10^ns^ | 6.04E+09^ns^ | 1.17E+09^ns^ | 8.21E+08^ns^ | 2.39E+09^ns^ | 2.79E+09^ns^ | 1.11E+09^ns^ | - | 1.61E+10^ns^ | 1.24E+11^ns^ | 4.35E+09^ns^ |
| UNAMEN | 4.41E+11^ns^ | 2.44E+10^ns^ | 4.89E+09^ns^ | 1.51E+09^ns^ | 3.15E+09^ns^ | 3.38E+08^ns^ | 2.04E+08^ns^ | 3.53E+09^ns^ | - | 2.68E+10^ns^ | 2.33E+11^ns^ | 5.77E+09^ns^ |
| TYPE | ns | ns | ns | ns | ns | ns | ns | ns | - | ns | ns | ns |
| ORIGIN | ns | ns | ns | ns | * | ns | ns | ns | - | ns | ns | ns |
| TYPE X ORIGIN | ns | ns | ns | ns | ns | ns | ns | ns | - | ns | ns | ns |

**Supplementary Table 2.** Kendall's rank correlation coefficients between prokaryotic orders in lettuce and wheat soils and ARG and MGE-gene absolute abundances grouped by antibiotic family or MGE category, followed by Bonferroni's multiple comparisons test.

|  | **Taxon/Antibiotic family or**  **MGE category** | **Aminoglycoside** | **β-lactamase** | **FCA** | **Integrase** | **MLSB** | **Multidrug** | **Sulfonamide** | **Tetracycline** | **Transposase** | **Vancomycin** |
| --- | --- | --- | --- | --- | --- | --- | --- | --- | --- | --- | --- |
| **Lettuce soil** | **Acidimicrobiales** |  |  |  |  |  |  |  |  |  | 0.45 |
|  | **Acidobacteria 1** |  | -0.47 |  | -0.41 |  |  |  |  |  | -0.40 |
|  | **Ardenticatenales** |  | -0.38 |  |  | -0.46 |  |  |  |  |  |
|  | **B1-7BS** |  |  |  | -0.39 |  |  |  |  |  |  |
|  | **BC-COM435** |  |  |  | -0.45 |  |  |  |  |  |  |
|  | **Bdellovibrionales** |  |  |  |  | -0.39 |  |  |  |  | -0.43 |
|  | **Blastocatellales** |  |  |  |  | -0.38 |  |  |  |  |  |
|  | **Burkholderiales** |  |  |  | -0.47 | -0.44 |  |  |  |  | -0.42 |
|  | **Caldilineales** |  |  |  |  |  |  | -0.38 |  |  |  |
|  | **Cellvibrionales** |  |  |  |  | -0.42 |  |  |  |  |  |
|  | **Chitinophagales** |  |  |  |  | -0.39 |  |  |  |  | -0.38 |
|  | **Chlorobiales** |  |  |  |  | -0.50 |  |  |  |  |  |
|  | **Chthoniobacterales** |  |  | -0.39 | -0.48 | -0.52 |  |  |  |  | -0.49 |
|  | **Cytophagales** |  |  |  |  | -0.42 |  |  |  |  | -0.39 |
|  | **Desulfurellales** |  |  |  |  |  |  |  |  |  | -0.41 |
|  | **Fibrobacterales** |  |  |  |  | -0.50 |  |  |  |  |  |
|  | **Flavobacteriales** |  |  |  |  | -0.40 |  |  | -0.40 |  | -0.43 |
|  | **Gaiellales** |  |  |  |  |  |  |  |  |  | 0.45 |
|  | **Halanaerobiales** |  |  |  | -0.38 |  |  |  |  |  |  |
|  | **HTA4** |  | -0.39 |  | -0.38 |  |  |  | -0.51 |  | -0.54 |
|  | **Legionellales** |  | -0.40 |  | -0.43 |  |  |  | -0.43 |  | -0.50 |
|  | **Leptospiriales** |  |  |  |  | -0.39 |  |  |  |  |  |
|  | **Micrococcales** |  |  |  |  | 0.41 |  |  |  |  | 0.41 |
|  | **Micromonosporales** |  |  |  |  |  |  |  |  |  | 0.42 |
|  | **mle1-8** |  |  |  |  |  |  |  |  | -0.42 |  |
|  | **NB1-j** |  |  |  |  |  |  |  |  |  | -0.46 |
|  | **Neisseriales** |  |  |  |  |  |  |  | -0.39 |  |  |
|  | **Obscuribacterales** |  | -0.42 |  | -0.50 | -0.39 |  |  |  |  | -0.46 |
|  | **Oceanospirillales** |  |  |  | -0.44 | -0.45 |  |  |  |  | -0.42 |
|  | **Oligoflexales** |  |  |  |  | -0.45 |  |  |  |  |  |
|  | **Opitutales** |  |  |  |  | -0.50 |  |  |  |  |  |
|  | **Planctomycetales** | -0.41 |  |  |  |  |  | -0.43 |  |  |  |
|  | **Propionibacteriales** |  |  |  |  |  |  |  |  |  | 0.39 |
|  | **Pseudonocardiales** |  |  |  |  | 0.39 |  |  | 0.41 |  | 0.43 |
|  | **Rhizobiales** |  |  |  |  | 0.45 |  |  | 0.41 |  |  |
|  | **Rickettsiales** |  |  |  |  | -0.43 |  |  |  |  |  |
|  | **Rubrobacterales** |  | 0.39 |  |  |  |  |  |  |  | 0.38 |
|  | **SAR324 clade** |  | -0.48 |  | -0.49 | -0.38 |  |  |  |  | -0.53 |
|  | **SC-I-84** |  |  |  |  |  |  | 0.38 |  |  |  |
|  | **Selenomonadales** |  |  |  |  |  |  | -0.41 |  |  |  |
|  | **Solirubrobacterales** |  |  |  | 0.38 | 0.46 |  |  | 0.39 |  | 0.59 |
|  | **Spartobacteria 1** |  |  |  |  |  |  |  |  |  | -0.40 |
|  | **Vampirovibrionales** |  | -0.47 |  | -0.45 |  |  |  |  |  | -0.38 |
| **Wheat soil** | **Puniceicoccales** |  |  |  | -0.45 |  |  |  |  |  |  |
|  | **47209** |  |  |  |  |  | -0.44 |  |  |  |  |
|  | **Kallotenuales** |  |  | -0.41 |  |  |  |  |  |  |  |
|  | **Synechococcales** |  |  |  |  |  |  | 0.45 |  |  |  |
|  | **Unknown Deltaproteobacteria order 4** |  |  |  |  | -0.44 |  |  |  |  |  |
|  | **Unknown Deltaproteobacteria order 5** |  |  |  | -0.44 |  |  |  |  |  |  |
|  | **Limnochordales** |  |  |  | 0.39 | 0.41 |  |  |  |  |  |
|  | **WN-HWB-116** | 0.46 |  |  |  |  |  |  |  | 0.42 |  |
|  | **S15A-MN16** |  |  |  |  |  |  | 0.39 |  |  |  |
|  | **CPla-3 termite group** |  |  |  |  |  |  | 0.41 |  |  |  |
|  | **Nostocales** |  |  |  |  |  |  | 0.38 |  |  |  |
|  | **JG30-KF-CM45** |  |  | -0.38 |  |  |  |  |  |  |  |
|  | **Tepidisphaerales** | 0.38 |  |  |  |  |  |  | 0.39 | 0.41 |  |
|  | **Xanthomonadales** |  |  | -0.47 |  |  |  |  |  |  |  |

**Supplementary Table 3.** Relative abundances of bacterial orders in lettuce soils and wheat soils (only those orders that showed statistically significant differences are presented). Different letters indicate significant (p<0.05) differences according to one-way ANOVA and Duncan`s multiple range test. ORG_AG: aged manure from organic farm; ORG_FRES: fresh manure from organic farm; ORG_SLU: slurry from organic farm; CONV_AG: aged manure from conventional farm; CONV_FRES: fresh manure from conventional farm; CONV_SLU: slurry from conventional farm; UNAMEN: unamended.

| **LETTUCE SOIL** | ORG_AG | ORG_FRES | ORG_SLU | CONV_AG | CONV_FRES | CONV_SLU | UNAMEN |
| --- | --- | --- | --- | --- | --- | --- | --- |
| **Acidobacteria group 22** | 4.71E-03^cd^ | 6.20E-03^b^ | 7.65E-03^a^ | 4.27E-03^cd^ | 5.01E-03^bc^ | 3.46E-03^d^ | 5.05E-03^bc^ |
| **C0119** | 8.06E-04^c^ | 1.03E-03^bc^ | 7.08E-04^c^ | 1.05E-03^bc^ | 9.25E-04^bc^ | 1.87E-03^a^ | 1.27E-03^b^ |
| **Chlorobiales** | 1.05E-03^b^ | 1.32E-03^ab^ | 1.07E-03^b^ | 1.60E-03^a^ | 9.38E-04^b^ | 1.50E-03^a^ | 5.20E-04^c^ |
| **Cytophagales** | 1.33E-02^d^ | 1.94E-02^ab^ | 2.02E-02^a^ | 1.79E-02^ab^ | 1.49E-02^cd^ | 1.67E-02^bc^ | 1.37E-02^cd^ |
| **KD4-96** | 1.65E-02^d^ | 1.94E-02^bcd^ | 1.51E-02^d^ | 1.81E-02^cd^ | 2.27E-02^bc^ | 2.45E-02^b^ | 3.17E-02^a^ |
| **Nitrososphaerales** | 1.54E-02^a^ | 1.66E-02^a^ | 1.02E-02^b^ | 1.27E-02^b^ | 1.15E-02^b^ | 1.58E-02^a^ | 1.62E-02^a^ |
| **Nitrospirales** | 2.96E-03^b^ | 4.60E-03^a^ | 3.44E-03^b^ | 3.36E-03^b^ | 2.90E-03^b^ | 3.31E-03^b^ | 3.04E-03^b^ |
| **Obscuribacterales** | 4.09E-04^ab^ | 3.87E-04^ab^ | 2.74E-04^bc^ | 3.16E-04^bc^ | 2.32E-04^cd^ | 4.57E-04^a^ | 1.16E-04^d^ |
| **Pseudonocardiales** | 5.99E-03^b^ | 5.55E-03^b^ | 5.74E-03^b^ | 5.72E-03^b^ | 6.08E-03^b^ | 5.62E-03^b^ | 8.76E-03^a^ |
| **SAR324 clade** | 2.22E-04^bc^ | 5.27E-04^a^ | 2.53E-04^bc^ | 2.12E-04^bc^ | 2.66E-04^bc^ | 3.28E-04^b^ | 1.47E-04^c^ |
| **SC-I-84** | 2.15E-02^a^ | 9.75E-03^b^ | 2.01E-02^a^ | 1.92E-02^a^ | 1.89E-02^a^ | 1.32E-02^b^ | 9.18E-03^b^ |
| **Solibacterales** | 3.37E-03^a^ | 3.57E-03^a^ | 3.46E-03^a^ | 3.43E-03^a^ | 3.54E-03^a^ | 2.79E-03^a^ | 1.72E-03^b^ |
| **Solirubrobacterales** | 2.85E-02^bc^ | 2.54E-02^bc^ | 2.29E-02^c^ | 2.98E-02^bc^ | 3.26E-02^b^ | 2.56E-02^bc^ | 4.09E-02^a^ |
| **Vampirovibrionales** | 3.74E-04^bc^ | 4.55E-04^b^ | 2.76E-04^cd^ | 3.74E-04^bc^ | 2.23E-04^cd^ | 6.34E-04^a^ | 2.13E-04^d^ |
| **Verrucomicrobiales** | 2.63E-03^c^ | 4.88E-03^a^ | 3.37E-03^bc^ | 3.10E-03^bc^ | 3.28E-03^bc^ | 3.81E-03^b^ | 2.71E-03^c^ |
| **WHEAT SOIL** | ORG_AG | ORG_FRES | ORG_SLU | CONV_AG | CONV_FRES | CONV_SLU | UNAMEN |
| **Desulfurellales** | 1.97E-02^ab^ | 1.85E-02^bc^ | 1.43E-02^d^ | 1.59E-02^cd^ | 1.79E-02^bc^ | 1.80E-02^bc^ | 2.26E-02^a^ |
| **Lineage IIb** | 1.19E-03^a^ | 6.37E-04^b^ | 6.65E-04^b^ | 9.68E-04^a^ | 1.09E-03^a^ | 4.86E-04^b^ | 6.94E-04^b^ |
| **Rhodospirillales** | 1.86E-02^c^ | 2.02E-02^bc^ | 2.22E-02^ab^ | 1.83E-02^c^ | 1.90E-02^c^ | 2.42E-02^a^ | 1.97E-02^bc^ |

**Supplementary Table 4.** Differences (based on Welch’s *t*-test) in ARG and MGE-gene absolute abundances in lettuce soils and wheat soils & in lettuce plants and wheat grains, according to treatments. L: lettuce samples; W: wheat samples. ORG_AG: aged manure form organic farm; ORG_FRES: fresh manure from organic farm; ORG_SLU: slurry from organic farm; CONV_AG: aged manure from conventional farm; CONV_FRES: fresh manure from conventional farm; CONV_SLU: slurry from conventional farm; UNAMEN: unamended. ns: no significant; *: p<0.05; **: p<0.01; ***: p<0.001.

|  | **Integrase** | | **Transposase** | | **Aminoglycoside** | | **β-lactamase** | | **FCA** | | **MLSB** | | **Sulfonamide** | | **Tetracycline** | | **Vancomycin** | **Multidrug** | |
| --- | --- | --- | --- | --- | --- | --- | --- | --- | --- | --- | --- | --- | --- | --- | --- | --- | --- | --- | --- |
| Treatment | Soil | Plant | Soil | Plant | Soil | Plant | Soil | Plant | Soil | Plant | Soil | Plant | Soil | Plant | Soil | Plant | Soil | Soil | Plant |
| ORG_AG | ns | ns | ns | ns | W>L* | W>L* | W>L** | ns | ns | ns | ns | ns | ns | ns | W>L* | W>L* | W>L** | W>L** | W>L*** |
| ORG_FRES | ns | ns | ns | ns | W>L** | ns | W>L** | ns | W>L* | W>L* | W>L* | ns | ns | ns | W>L** | ns | W>L* | ns | ns |
| ORG_SLU | ns | W>L* | ns | ns | W>L* | W>L* | W>L* | W>L* | ns | ns | ns | ns | ns | ns | W>L* | W>L* | ns | ns | W>L* |
| CONV_AG | ns | W>L* | ns | ns | ns | W>L* | W>L* | ns | ns | ns | ns | ns | ns | ns | ns | ns | ns | ns | W>L* |
| CONV_FRES | ns | W>L* | ns | ns | W>L* | ns | W>L** | ns | ns | ns | ns | ns | ns | ns | W>L* | ns | W>L* | ns | ns |
| CONV_SLU | ns | W>L* | ns | ns | W>L** | ns | W>L** | ns | ns | ns | ns | ns | ns | ns | ns | W>L* | ns | ns | W>L* |
| UNAMEN | ns | ns | ns | ns | ns | ns | ns | ns | ns | ns | ns | ns | ns | ns | ns | ns | ns | ns | ns |
